# Case Report: Native aortic valve *Listeria monocytogenes* endocarditis in an adult with Evans syndrome

**DOI:** 10.3389/fimmu.2026.1772213

**Published:** 2026-02-25

**Authors:** Chen-Yu Wei, Hsiang-Chun Lee, Li-Teh Liu, Pei-Chi Yen, Chen-Hsuan Lin, Jih-Jin Tsai

**Affiliations:** 1 Division of Infectious Diseases, Department of Internal Medicine, Kaohsiung Medical University Hospital, Kaohsiung, Taiwan; 2 School of Medicine, College of Medicine, Kaohsiung Medical University, Kaohsiung, Taiwan; 3 Division of Cardiology, Department of Internal Medicine, Kaohsiung Medical University Hospital, Kaohsiung Medical University, Kaohsiung, Taiwan; 4 Lipid Science and Aging Research Center, College of Medicine, Kaohsiung Medical University, Kaohsiung, Taiwan; 5 Department of Internal Medicine, Kaohsiung Medical University Gangshan Hospital, Kaohsiung Medical University, Kaohsiung, Taiwan; 6 Department of Medical Laboratory Science and Biotechnology, College of Medical Technology, Chung Hwa University of Medical Technology, Tainan, Taiwan; 7 Division of Gastroenterology, Department of Internal Medicine, Kaohsiung Medical University Hospital, Kaohsiung Medical University, Kaohsiung, Taiwan; 8 Tropical Medicine Center, Kaohsiung Medical University Hospital, Kaohsiung, Taiwan

**Keywords:** bacteremia, case report, corticosteroids, Evans syndrome, immunosuppression, infective endocarditis, listeria monocytogenes

## Abstract

**Background:**

*Listeria monocytogenes* is an uncommon cause of infective endocarditis but is associated with a high morbidity and mortality rate in immunocompromised hosts. Evans syndrome, typically treated with prolonged high-dose corticosteroids and additional immunosuppressants, may predispose patients to severe opportunistic infection.

**Case presentation:**

A 44-year-old man with Evans syndrome on prednisolone and azathioprine, and with recent disseminated cryptococcosis, presented with acute right upper quadrant abdominal pain after ingesting oyster-containing street food. The patient did not exhibit any fever or neurological symptoms. Laboratory tests showed leukocytosis, thrombocytopenia, elevated inflammatory markers, and mild renal and hepatic dysfunction. Abdominal computed tomography (CT) findings were unremarkable. Empirical cefoperazone/sulbactam was initiated for suspected intra-abdominal infection and escalated to meropenem because of clinical deterioration. Blood cultures subsequently grew *Listeria monocytogenes*, prompting a de-escalation to high-dose intravenous ampicillin, resulting in rapid symptomatic improvement. Transthoracic and transesophageal echocardiography revealed multiple small, oscillating vegetations on the native aortic valve, consistent with infective endocarditis, without heart failure or embolic complications. The patient completed a 6-week course of ampicillin therapy. Follow-up transesophageal echocardiography showed partial resolution of the vegetations. The corticosteroids and azathioprine were gradually tapered and then discontinued, with sustained remission of Evans syndrome and no recurrence of invasive infections.

**Conclusion:**

This case likely represents the first reported instance of native-valve *Listeria monocytogenes* endocarditis in a patient with Evans syndrome. It highlights prolonged immunosuppression in Evans syndrome as a potential risk context for invasive listeriosis with cardiac involvement. Early blood culture, routine echocardiographic evaluation for *Listeria* bacteremia, and timely targeted therapy are essential for optimizing patient outcomes.

## Introduction

*Listeria monocytogenes* is a Gram-positive, facultative intracellular coccobacillus that is widely distributed in the environment, including water, soil, vegetation, and animal feces. Contaminated food products constitute the primary source of human infection (1). Human listeriosis is relatively uncommon, with an estimated global incidence of 0.1–10 cases per million people annually. However, it predominantly affects high-risk hosts and is frequently associated with severe and invasive syndromes (2). Clinical manifestations range from self-limited febrile gastroenteritis to invasive disease such as bacteremia, neurolisteriosis, and, rarely, infective endocarditis (1, 3).

Endocarditis caused by *Listeria monocytogenes* represents a particularly uncommon yet life-threatening form of invasive listeriosis. Published series indicate that endocardial involvement occurs in only a small fraction of listeriosis cases but is associated with mortality rates approaching 30–50%, largely due to embolic complications and heart failure. Fewer than 200 cases of listerial endocarditis with detailed clinical data have been reported, most occurring in elderly patients or those with significant comorbidities, prosthetic valves, and underlying structural heart diseases (3, 4). Immunological status is a key determinant of disease severity; in addition to traditional risk groups such as pregnancy, advanced age, malignancy, diabetes mellitus, and chronic organ dysfunction, the increasing use of corticosteroids and biologic therapies has expanded the population at risk for severe listeriosis (5, 6).

Evans syndrome is a rare autoimmune disorder characterized by the concomitant or sequential occurrence of immune thrombocytopenia and autoimmune hemolytic anemia, with autoimmune neutropenia present in some cases. Management commonly requires prolonged high-dose corticosteroid and additional immunosuppressive agents (7). Such treatment strategies may predispose patients to opportunistic infections; however, the association between Evans syndrome and invasive listeriosis, specifically listerial endocarditis, has rarely been described. Here, we report a case of native aortic valve *Listeria monocytogenes* endocarditis in a middle-aged man with Evans syndrome who was receiving high-dose corticosteroid and azathioprine therapy, and discuss the evolving spectrum of host-related risk factors for listerial endocarditis, despite the continued rarity of invasive listeriosis.

## Case description

A 44-year-old man, an engineer with a history of bilateral glaucoma and hypertension, was diagnosed six months earlier with Evans syndrome, presenting with immune thrombocytopenia and autoimmune hemolytic anemia. After initial stabilization, he was discharged on oral prednisolone 20 mg and azathioprine 50mg daily. Four months before the current admission, he developed fever and headache and was diagnosed with disseminated cryptococcosis complicated by cryptococcal meningitis and a left pulmonary cryptococcoma. During that prior hospitalization, transthoracic echocardiography was performed and did not demonstrate valvular vegetations or structural abnormalities. He received induction therapy with amphotericin B and flucytosine followed by high-dose fluconazole consolidation. During that hospitalization, prednisolone was escalated to 60 mg daily due to progression of Evans syndrome.

Approximately one month after completing the induction antifungal therapy and hospital discharge, he presented to the emergency department with a two-day history of acute, stabbing right upper quadrant abdominal pain that began shortly after consuming oyster-containing street food. The pain worsened with positional changes and deep inspiration but improved with standing and rest. Associated symptoms included dyspnea and constipation; however, he denied fever, chills, headache, nausea, vomiting, jaundice, melena, or diarrhea. On presentation, his temperature was 35.6 °C, blood pressure 171/129 mmHg, heart rate 120 beats per minute, respiratory rate 22 breaths per minute, and oxygen saturation 95% on room air; supplemental oxygen was initiated due to dyspnea. Abdominal examination revealed right upper quadrant tenderness without Murphy’s sign, rebound tenderness, or guarding, and bowel sounds were normal.

Laboratory tests showed leukocytosis (white blood cell count 10,990/µL), hemoglobin of 12.5 g/dL, and thrombocytopenia (platelet count 89,000/µL). Serum alanine aminotransferase was mildly elevated at 62 IU/L, creatinine was 1.31 mg/dL, and inflammatory markers were increased (C-reactive protein 8.27 mg/dL; D-dimer 3.78 mg/L). Troponin I was within the normal range. Non-contrast abdominal computed tomography revealed no evidence of cholecystitis, biliary obstruction, abscess, or other intra-abdominal pathology. Two sets of blood cultures were obtained, and empirical intravenous cefoperazone/sulbactam was initiated for suspected sepsis.

Despite antimicrobial therapy, the patient’s abdominal pain worsened on hospital day 5, prompting the escalation of intravenous meropenem for possible resistant infection. Subsequent blood cultures grew *Listeria monocytogenes*, leading to notification of the national Centers for Disease Control and de-escalation to targeted therapy with intravenous ampicillin (2g every 4h). Meropenem was discontinued after repeat cultures failed to identify Gram-negative organisms, and the patient’s abdominal pain improved substantially within days of starting ampicillin. In the absence of neurological symptoms, lumbar puncture was deferred, and echocardiographic evaluation was performed, given the documented *Listeria* bacteremia.

**Figure 1:**
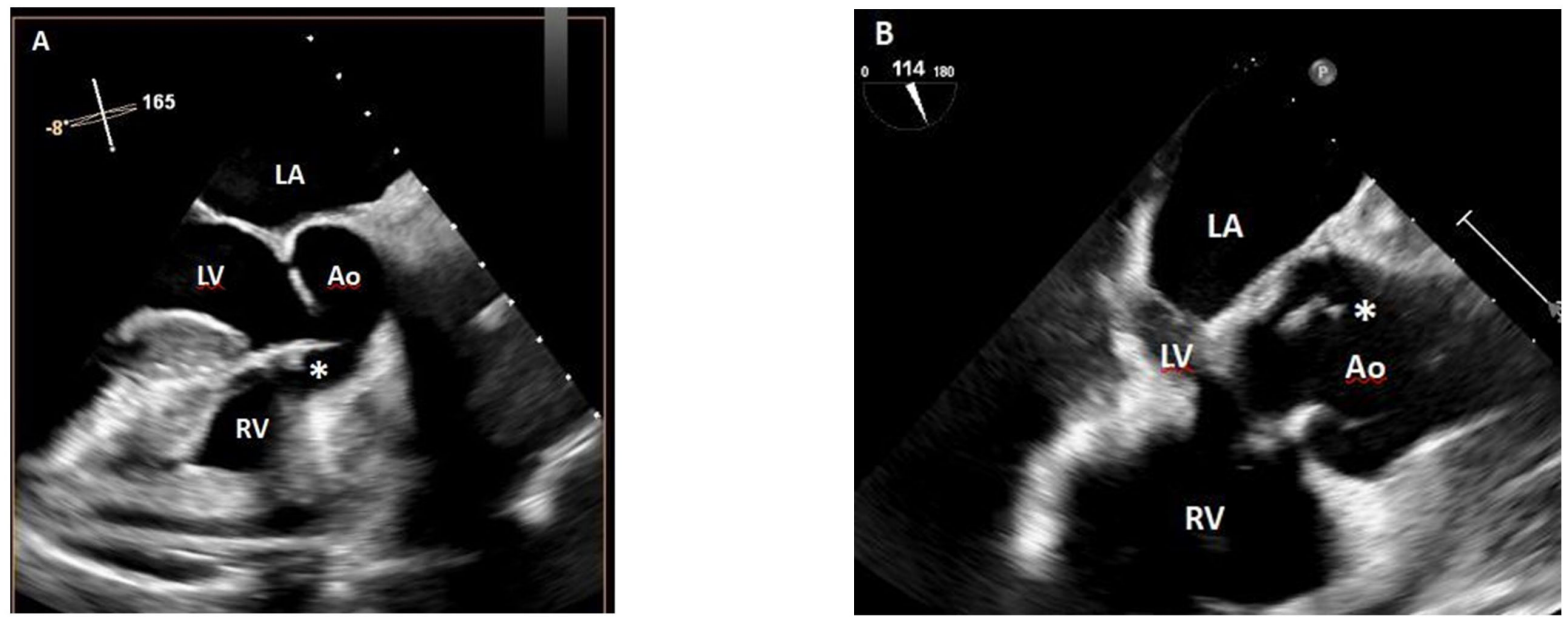
Transesophageal echocardiography (TEE) **(A)** reveals small vegetations (0.19-0.46 cm) on the aortic valve, and follow-up TEE **(B)** shows partially resolved oscillating vegetations (0.17-0.29 cm).

Transthoracic echocardiography revealed aortic valve thickening with small mobile vegetations measuring approximately 0.1–0.2 cm, raising suspicion of infective endocarditis. Transesophageal echocardiography confirmed multiple oscillating vegetations on the native aortic valve, measuring 0.19–0.46 cm, and without significant valvular dysfunction or perivalvular extension (**Figure 1A**). The patient remained hemodynamically stable, and no clinical or radiologic evidence of embolic events was observed. A repeat transesophageal echocardiogram at week 5 of therapy showed partial resolution of the vegetations, now measuring 0.17–0.29 cm, with preserved left ventricular function and no new valvular lesions (**Figure 1B**).

The patient completed a 6-week course of intravenous ampicillin without adverse events, and follow-up blood cultures remained sterile. Additional evaluations, including positron emission tomography/computed tomography (PET-CT), autoimmune serologic marker testing, and bone marrow examination, excluding malignancy or alternative autoimmune disorders. He was discharged in stable condition with a planned follow-up at the rheumatology/immunology and infectious diseases clinics. Prednisolone and azathioprine were gradually tapered with close monitoring and finally totally discontinued after 14 months. Evans syndrome remained in sustained remission, and no recurrence of invasive fungal or bacterial infections was observed during the 10-month follow-up period.

## Discussion

This case illustrates several clinically important aspects of *Listeria monocytogenes* infection in an immunocompromised host, including an atypical presentation dominated by right upper quadrant abdominal pain, the development of native aortic valve endocarditis, and the contributory risk profile associated with Evans syndrome, which was managed with prolonged high-dose corticosteroids and azathioprine. In immunocompetent individuals, *Listeria* infection most often manifests as self-limited gastroenteritis; however, impaired cell-mediated immunity facilitates intracellular survival, translocation across the intestinal mucosa, and subsequent bacteremia with seeding of distant organs, such as the central nervous system and heart valves (8). Accordingly, invasive listeriosis is strongly associated with host factors such as advanced age, pregnancy, diabetes mellitus, malignancy, liver cirrhosis, end-stage renal disease, and increasing exposure to immunosuppressive and biologic therapies (9).

Infective endocarditis represents a rare but clinically significant manifestation of invasive listeriosis. Published series and literature reviews have identified fewer than 100–200 well-characterized cases worldwide, with reported mortality rates of approximately 30%, which is higher in native-valve than prosthetic-valve disease. Embolic complications, particularly those involving the central nervous system and peripheral vasculature, as well as progressive valvular destruction, significantly contribute to this high mortality rate, underscoring the importance of early recognition, echocardiographic assessment, and prompt initiation of appropriate antimicrobial therapy (3, 4). In the systematic review by Kypraiou et al., 100 cases of Listeria endocarditis were identified; 54.8% occurred on prosthetic valves and the remainder on native valves, and most patients were elderly with significant comorbidities or structural heart disease (4). Our patient differs in that he was relatively young and had no known pre-existing valvular abnormality, but had profound immunosuppression due to Evans syndrome and its treatment, a risk profile that has not been specifically highlighted in previous series. Our case had no embolic complications of infective endocarditis compared with the prior cases (4). It might be partly due to early detection and timely antibiotics treatment, so only a small vegetation (0.19–0.46 cm) was found in the transesophageal echocardiography without valvular dysfunction. In Taiwan, national surveillance data indicate that invasive listeriosis remains uncommon, with approximately 150 cases reported annually in recent years, and listerial endocarditis has not been previously documented in Taiwan, making this case likely the first reported (10).

Evans syndrome is a rare autoimmune cytopenia characterized by immune thrombocytopenia and autoimmune hemolytic anemia, with autoimmune neutropenia present in some patients, and typically necessitates long-term immunosuppressive management. Current consensus recommendations support the initiation of treatment with high-dose corticosteroids (approximately 1 mg/kg/day of prednisone), followed by the addition of immunosuppressants or biologics, such as azathioprine or rituximab, in cases of relapsing or refractory disease (7). Such regimens substantially impair host defenses against intracellular pathogens and opportunistic fungi, as illustrated by this patient’s recent disseminated cryptococcosis and subsequent invasive listeriosis while receiving escalating doses of prednisolone and azathioprine.

Reports specifically linking Evans syndrome to *Listeria monocytogenes* infection are limited. A prior case described a hepatic abscess due to Listeria monocytogenes in a patient with Evans syndrome who later underwent splenectomy, highlighting the potential for severe focal infections in this population (11). The present case extends this association to include native valve endocarditis, suggesting that Evans syndrome, particularly when managed with prolonged high-dose corticosteroids and additional immunosuppressive therapy, should be recognized as a non-traditional but significant risk factor for invasive listeriosis with cardiac involvement. Clinicians caring for patients with Evans syndrome who present with systemic symptoms suggestive of sepsis should maintain a high index of suspicion for *Listeria monocytogenes*, especially in the context of high-risk dietary exposures such as unpasteurized dairy products or ready-to-eat food items.

From a therapeutic standpoint, high-dose ampicillin remains the cornerstone of treatment for invasive listeriosis, with aminoglycoside combination therapy often considered to achieve synergistic bactericidal activity. However, the optimal regimen for listerial endocarditis has not been definitively established. Retrospective analyses suggest that prolonged β-lactam monotherapy may be sufficient in selected patients with native-valve disease who remain clinically stable, whereas combination therapy and early surgical intervention are more frequently employed in patients with prosthetic valve disease or complicated infections (3). In this patient, clinical stability, absence of heart failure or embolic events, and serial echocardiographic evidence of vegetation regression supported a conservative, medically managed approach with 6 weeks of intravenous ampicillin, resulting in microbiological cure and favorable long-term outcomes.

Finally, this case emphasizes the diagnostic value of early echocardiography in patients with *Listeria monocytogenes* bacteremia, even in the absence of overt cardiac symptoms, particularly in those with severe immunosuppression. The atypical initial presentation with isolated abdominal pain, unremarkable abdominal imaging, and absence of fever could have delayed the recognition of invasive listeriosis or endocarditis; however, prompt blood cultures, awareness of the patient’s immunological status, and echocardiographic evaluation allowed identification of clinically silent aortic valve vegetations, highlighting the need to recognize atypical presentations of *Listeria* infection in immunocompromised individuals.

## Take-away lessons

Prolonged immunosuppression for Evans syndrome can create a vulnerable context for atypical, invasive infections such as native-valve Listeria monocytogenes endocarditis, even in relatively young patients without structural heart disease. This case, likely the first reported native-valve *Listeria monocytogenes* endocarditis in a patient with Evans syndrome, and among the first from Taiwan. In immunocompromised patients presenting with non-specific symptoms such as isolated abdominal pain and no fever, clinicians should maintain a high index of suspicion for invasive listeriosis, particularly after high-risk food exposures, and obtain prompt blood cultures. Listeria bacteremia in immunosuppressed hosts warrants early echocardiographic assessment and rapid initiation of Listeria-directed antibiotics once identified, as timely, targeted therapy may obviate the need for surgery and improve outcomes. Systematic re-evaluation of corticosteroid and immunosuppressant dosing after severe opportunistic infections is crucial, as careful tapering may restore immune control while maintaining remission of Evans syndrome.

## Patient perspective

“Looking back, I did not realize how vulnerable my immune system had become while I was taking high-dose steroids and azathioprine for Evans syndrome. When the abdominal pain started, I was surprised that I never had a fever and thought it was just something I had eaten, not a serious infection involving my heart.

I was anxious when I learned that bacteria from contaminated food had entered my bloodstream and affected my aortic valve, but I felt reassured by the close monitoring, repeated heart scans, and clear explanations from the medical team. Completing the long course of intravenous antibiotics and gradually stopping the immunosuppressive drugs gave me confidence that my body could recover, and I am now more cautious about my diet and more aware that treatment for autoimmune disease can increase my risk for severe infections.”

## Data Availability

The original contributions presented in the study are included in the article/supplementary material. Further inquiries can be directed to the corresponding author.
